# Percutaneous Cannulation of the Axillary Artery: Does the Position of the Brachial Plexus Correlate With Bony Landmarks That Could Be Used to Reduce the Risk of Complications?

**DOI:** 10.7759/cureus.67926

**Published:** 2024-08-27

**Authors:** Sourabh Jadhav, Ashley Stephen

**Affiliations:** 1 Anatomical Sciences, School of Medicine, Keele University, Keele, GBR; 2 General Surgery, Tameside General Hospital, Manchester, GBR; 3 Anatomy, School of Medicine, Keele University, Keele, GBR

**Keywords:** peripheral arterial disease (pad), axillary artery anatomy, cadaveric dissection, bony landmarks for cannulation, safe arterial cannulation, endovascular procedures, brachial plexus injury, cadaveric research, axillary artery access

## Abstract

Introduction

Endovascular surgery is an innovative way of carrying out procedures such as transcatheter aortic valve insertion where the femoral artery is commonly used as an access point. Conditions like peripheral arterial disease can make endovascular procedures challenging when atherosclerotic plaques compromise the integrity of lower limb vessels. An alternative access point for these patients is required. Access through the axillary artery has been proposed; however, the close proximity of the brachial plexus introduces a risk of neural complications. This study aims to find an anatomical or bony landmark(s) to help identify an area of safety on the axillary artery that can be used to gain access.

Materials and methods

Nine cadavers were used in the study and five parameters were measured using the acromion and coracoid processes as bony landmarks. The 1st parameter measured the distance between the acromion and the coracoid process. The 2nd parameter was the diameter of the axillary artery taken at a plane extending from the acromion to the coracoid process - now defined as the coracoacromial plane. The 3rd measurement was the distance between the coracoid process and the midpoint of the axillary artery diameter taken at the above plane; it is proposed this will form a safe point on the axillary artery. The 4th parameter measured was the distance between the safe point on the axillary artery and the median nerve. The 5th parameter was the distance between the safe point and the thoracoacromial trunk. Measurements were taken using digital callipers and were recorded for both sides of the cadaver except for one. Using the data from the measurements, an area of safety was calculated and statistical analysis was carried out using Student's t-test and Pearson’s correlation to look for significant differences between the left and right sides.

Results

The mean distance from the safe point of the axillary artery to the median nerve was 23.25 mm on the left and 27.10 mm on the right. The p-value was 0.7, which indicated no significant differences between both sides. The mean distance between the safe point and the thoracoacromial trunk was 11.31 mm on the left and 13.21 mm on the right. The p-value was 0.24, indicating no significant differences between both sides. The mean area of safety was larger on the right side with an area of 184.37 mm and smaller on the left side with an area of 158.93 mm. The p-value was 0.62, which indicated no significant differences between both sides. There was no clear relationship between the distance from the acromion to the coracoid process compared to the distance between the acromion and a defined safe point on the axillary artery. This was confirmed using a Pearson’s correlation test, which resulted in a p-value of 0.53 on the left and 0.93 on the right. These values were above the critical value, suggesting no correlation.

Conclusion

The acromion and the coracoid process are important bony landmarks that can be used to define the coracoacromial plane that traverses the axillary artery whereby avoiding the cords of the brachial plexus, the median nerve as well as the thoracoacromial trunk. Implementing this approach to define a safe vascular access point on the axillary artery could minimise complications like brachial plexus injuries. Further studies on a larger sample size using radiological methods may need to be carried out to help increase confidence in these preliminary cadaveric findings.

## Introduction

A crucial aspect of surgical research relies on ensuring surgery is as safe and as effective as possible. One facet of this is the evolution of minimally invasive endovascular surgery that provides an efficient way of treating many vascular pathologies, including vascular aneurysms or arterial dissections, and is often incorporated in cardiac surgical procedures, such as transcatheter aortic valve insertion (TAVI) or percutaneous coronary intervention (PCI). All endovascular procedures require an access point in the form of a blood vessel through which a surgeon can insert a catheter that is advanced up to the area of interest [1]. The most common access point for these procedures is the femoral artery due to its palpable and consistent location at the mid-inguinal point [2]. A small incision is made over the artery, which is then cannulated ready for the catheter to be inserted. This avoids the need for a larger scar and decreases the incidence of post-operative complications by reducing the risk of infection. Although the most common point of vascular access remains the femoral artery, there are certain instances where cannulation of the femoral artery may not be possible. Peripheral arterial disease (PAD) is a condition where the tunica intima (internal endothelial lining) of elastic or muscular arteries undergo fibrotic changes that narrow the lumen and weaken the vessel, thus leading to difficulty gaining endovascular access [3]. Furthermore, hostile iliac anatomy can also be a limiting factor when placing a cannula in the femoral artery [4]. This can lead to a proportion of patients not being able to undergo certain endovascular procedures [2]. To overcome this issue, an alternative access point that can be used is the axillary artery [5]. Both the femoral and axillary approaches come with various procedure-related complications; however, an important consideration to make for using the axillary artery is its close proximity to the brachial plexus, which has a risk of getting damaged during the initial insertion of the cannula.

Therefore, trying to determine an anatomical landmark that can be used to identify a safe area through which the axillary artery can be cannulated whilst reducing the risk of complications would be beneficial.

Axillary artery anatomy

The axillary artery starts at the level of the first rib by continuing from the subclavian artery. It courses anterior to the glenohumeral joint before crossing the lower border of teres major muscle where it continues into the anterior compartment of the arm as the brachial artery. The sensory and motor innervation supplying the upper extremity is intimately associated with the axillary artery, as the cords of the brachial plexus are named according to their relationship running alongside this vessel.

As the axillary artery travels between the first rib and the lower border of teres major, it is categorised into three different sections. The pectoralis minor muscle is used as a point of reference to define the second part of the artery and its respective branches. The first part of the axillary artery gives off a superior thoracic branch. The second part has two branches: the lateral thoracic artery and a prominent thoracoacromial trunk, which divides into four subsequent branches. The third part gives rise to the subscapular, anterior circumflex humeral and posterior circumflex humeral branches.

Although the origin of the axillary arteries is constant in terms of being a continuation of a subclavian artery, differences exist where the subclavian arteries arise on the left and the right side. The right subclavian originates from the brachiocephalic trunk, which is a more proximal branch from the arch of the aorta, whereas the left subclavian artery is a direct branch from further along the arch of the aorta.

## Materials and methods

Ethical approval

Prior to this project being carried out, it was reviewed by the Anatomical Research Group (ARG) and was subsequently given ethical approval by the ARG ethics subcommittee. The research was carried out in its entirety at Keele University’s School of Medicine Anatomical Facilities, under the guidance of the Human Tissue Authority (HTA) license #12190 (Section 16;2;c of the Human Tissue Act 2004).

Cadaveric and photographic permissions

The study was carried out on nine cadavers that were consented for scientific research and were donated to the School of Medicine at Keele University in accordance with the Human Tissue Act of 2004 approved by the HTA. The cadaveric aspects of the study took place in its entirety at Keele Anatomy and Surgical Training Centre (KASTC), which is a licensed premises. The cadaveric photography present in this section was captured with a Canon EOS 700D camera (Canon Inc., Tokyo, Japan) making sure that the donors had granted image permission prior to taking photographs.

Study sample

There were no inclusion or exclusion criteria that were applied in this study; however, information on the cause of death was obtained to help determine whether any underlying pathologies may have affected the results. Table 1 summarises the demographic data of each cadaver, including information on biological sex. To help maintain anonymity, the respective age and primary cause of death for each donor have been generalised for the purposes of this article. Information regarding patient ethnicity was not available.

**Table 1:** A summary of the demographic data of the cadavers used in this study. Data on ethnicity were not available. The causes of death of the patients were as follows: 1: neurodegenerative disease; 2: cancer; 3: respiratory disease; 4: cancer; 5: old age; 6: cardiovascular disease; 7: cardiovascular disease; 8: cancer; 9: cancer.

Cadaveric specimen	Biological sex	Age at death (converted into range)
1	Male	70-74 years
2	Female	90-94 years
3	Male	75-79 years
4	Male	70-74 years
5	Male	85-89 years
6	Male	80-84 years
7	Female	90-94 years
8	Male	80-84 years
9	Female	85-89 years

Statistical analysis

A paired two-tailed Student's t-test was selected to determine the significance of variation between the data that were collected. The tests were carried out using Microsoft Excel (Microsoft Corporation, Redmond, WA), with a data analysis add-on used for statistical calculations. A p-value of less than 0.05 was considered significant for this study. The results from cadaver 1 were not included in any of the statistical calculations since only the data from one (left) side of the cadaver were available.

Cadaveric embalming process

Eight of the nine cadavers were kindly donated to the university three years prior to the commencement of this study with one (cadaver 1) being donated five years prior. Each of the cadavers used in this project was embalmed and cared for by trained anatomy technicians at KASTC. The following method was used to do this: an incision in the inner thigh was made to locate the femoral artery in the femoral triangle, being careful not to damage any veins nearby. Once the artery was located, an aneurysm hook was used to raise the artery and a small incision in the artery was made. Two cannulas were then put inside the artery, one facing up and the other facing down. The cannulas were then attached to an embalming machine, which first pumps saline around the body. Embalming fluid was then pumped through the up-facing cannula first, followed by the down-facing cannula. The fluid is made up using the following constituents: 71.8% ethanol, 10% phenol, 3.8% methanol, 6% glycerol, 1.6% formaldehyde, and 6.8% water. The length of time attached to the pump varied (based on experience and technical judgement) but once enough embalming fluid was used, the cannulas were removed, and the incisions were stitched up.

Cadaveric dissection

In regard to exposing the axillary artery and surrounding anatomical structures, eight of the nine cadavers had been previously dissected by 1st and 2nd-year medical students (cadavers 2-9) at Keele University and one cadaver (cadaver 1) was dissected by an experienced lecturer of anatomy at Keele University’s Medical School.

**Figure 1:**
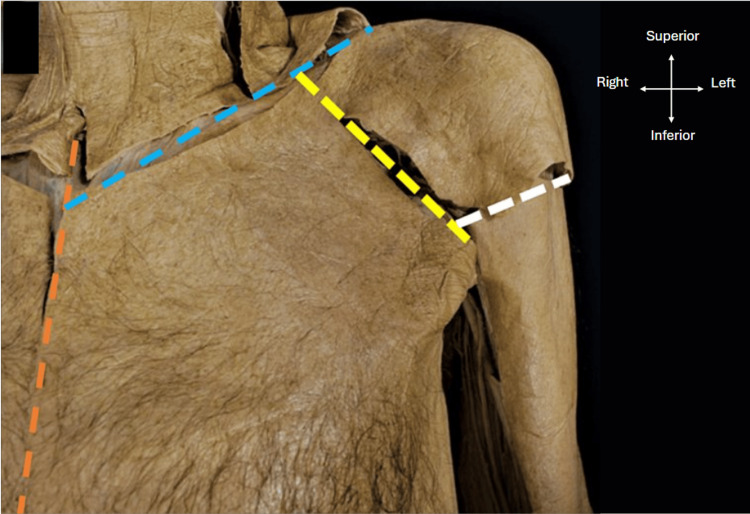
Skin incisions for the dissection. Image showing the incisions made during dissection of the cadavers with a midline incision (orange dashed line), a sub-clavicular incision (blue dashed line), an incision in the deltopectoral groove (yellow dashed line), and an incision around the surgical neck of the humerus (white dashed line). An orientation grid has been provided.

Figure 1 shows the skin incisions made during the dissection process. Firstly, a scalpel was used to make a midline incision extending from the jugular notch to the xiphoid process of the sternum (orange dashed line). A sub-clavicular incision was then carried out extending from the midline to the lateral aspect of the acromion process (blue dashed line). An incision overlying the deltopectoral groove was also made (yellow dashed line) extending from the coracoid process to the proximal humerus below the greater and lesser tuberosities. A final incision was made from the incision of the deltopectoral groove around the surgical neck of the humerus (white dashed line).

**Figure 2:**
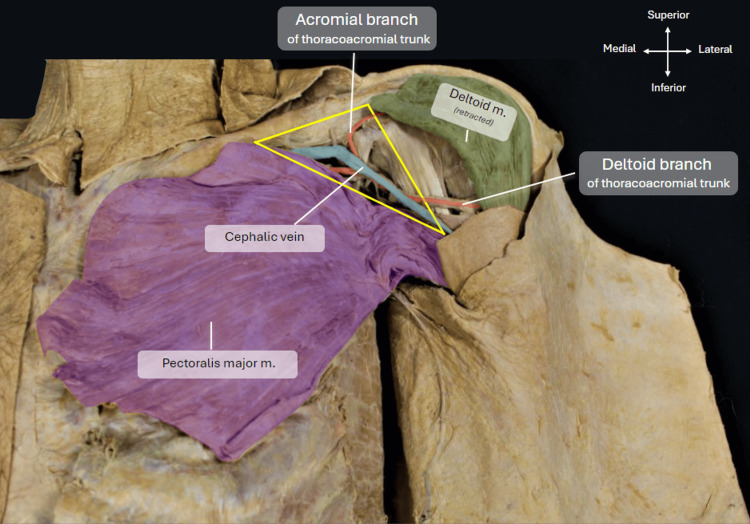
Structures below skin incisions. Image showing the structures underneath the incisions that were made with the skin and subcutaneous fat being reflected out of the way. The deltopectoral triangle is outlined in the yellow triangle with the cephalic vein and the acromial branch of the thoracoacromial trunk being visible in the triangle. The pectoralis major muscle has also been highlighted in purple.

Once the incisions were made, the skin and superficial fascia were carefully dissected and reflected back to reveal the underlying muscles, which can be seen in Figure 2 highlighted in purple and green. Blunt dissection was continued into the subclavicular region to remove fat and fascia around the superior margin of the pectoralis major muscle and medial border of the deltoid muscle to identify the contents of the deltopectoral triangle, as outlined in yellow in Figure 2. The cephalic vein was followed deep into the axillary sheath to locate where it drains into the axillary vein. The pectoralis major muscle was detached from its clavicular, sternal, and costal attachments with the origin of the muscle (lateral lip of the intertubercular sulcus) and neurovascular structures (medial and lateral pectoral nerves as well as pectoral branches of the thoracoacromial trunk) still intact.

**Figure 3:**
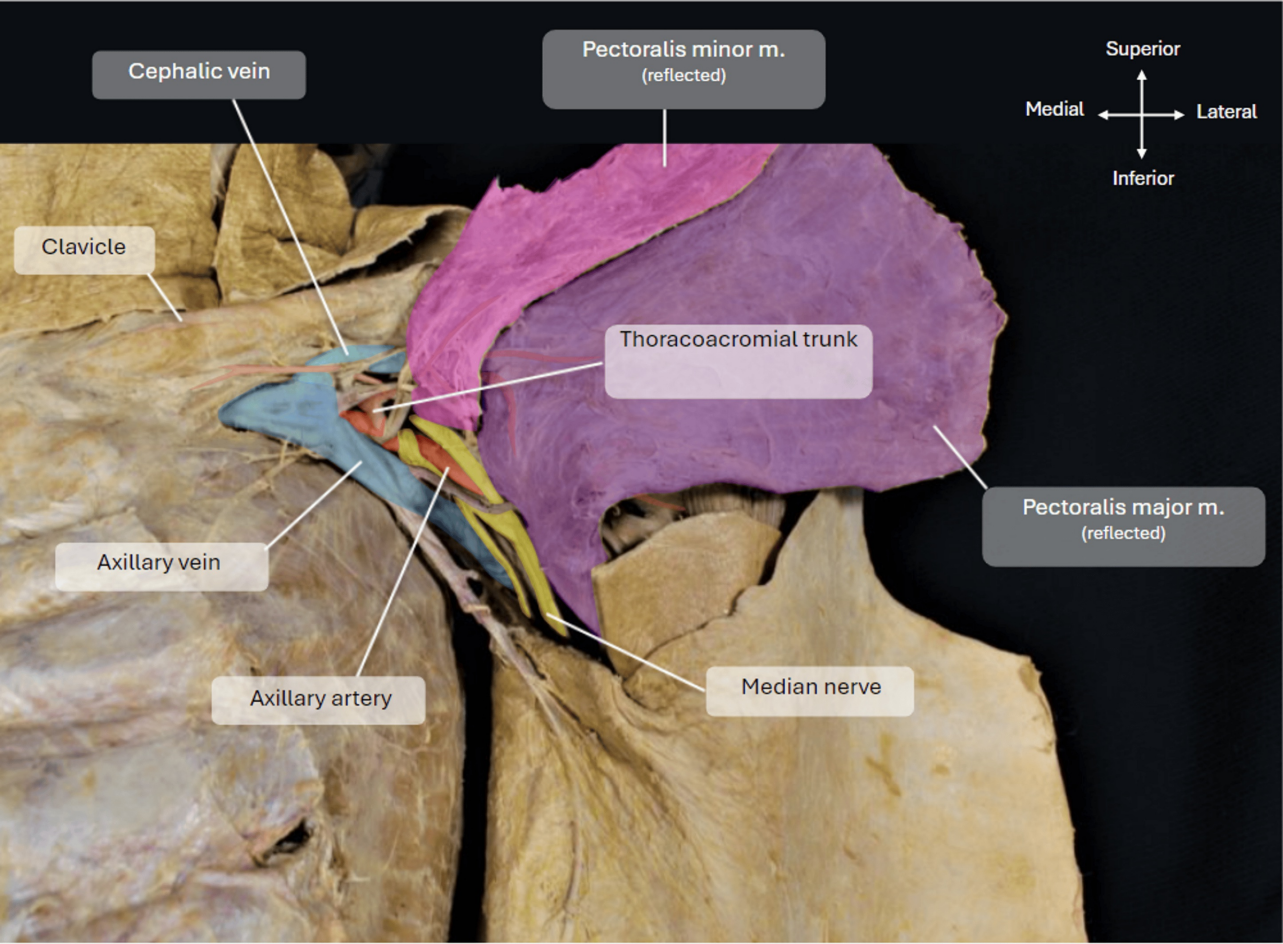
Dissection of pectoralis major and minor. Image showing the pectoralis major and minor dissected and reflected away from the chest wall to expose the axillary artery, vein, and brachial plexus underneath.

The pectoralis minor muscle was dissected away from its costal attachments whilst keeping it attached laterally to an important bony landmark for this project, the coracoid process. The pectoralis minor muscle also acts as a significant anatomical landmark to define the three different parts of the axillary artery: proximal to the medial border of the pectoralis minor muscle is considered the first part, underneath the muscle belly lies the 2nd part, and distal to the lateral border lies the 3rd part of the axillary artery. The axillary sheath encasing the axillary vein (highlighted in blue) and artery (highlighted in red) was further dissected to reveal the brachial plexus (highlighted in yellow) and branches of the respective parts of the axillary artery, with particular focus on the thoracoacromial trunk. Figure 3 shows pectoralis major and minor reflected laterally to reveal the axillary artery and vein below.

**Figure 4:**
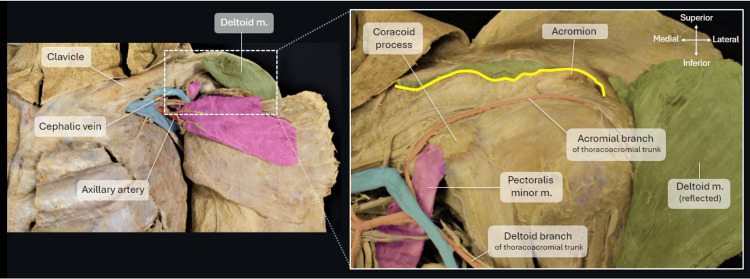
Dissection of the deltoid. Photograph showing the incisions used (yellow line) to detach the deltoid muscle away from its attachments to the clavicle and acromion. The highlighted section on the image shows a higher magnification of the left anterior shoulder with the deltoid reflected to visualise and access the acromion and coracoid process of the scapula.

Blunt dissection was used to mobilise the anterior border of the deltoid muscle, which was subsequently reflected by incising the proximal attachments to the clavicle and acromion process superiorly. The yellow line in Figure 4 represents the cut edge where the deltoid muscle was detached to expose the edge of the coracoacromial ligament and humeral head. Both the acromion and the coracoid process were used as palpable bony landmarks to carry out various measurements that are detailed in the measurements section.

Parameters to be measured

Five parameters were measured in this study and are outlined as follows: parameter 1: distance between the acromion process and coracoid process. Parameter 2: diameter of the axillary artery taken at the plane extending from the acromion to the coracoid process, now defined as the coracoacromial plane. Parameter 3: distance between the coracoid process and the midpoint of the axillary artery diameter taken at the coracoacromial plane. The midpoint of the axillary artery at this location will now be referred to as the safe point. Parameter 4: distance between the safe point on the axillary artery and the median nerve. Parameter 5: distance between the safe point on the axillary artery and the thoracoacromial trunk.

The above measurements were taken from both the left and the right sides for cadavers 2-9; however, only the left side was measured for cadaver 1 since this was also used as a prosection body for teaching demonstrations. Using the measurements above, an area of safety around the safe point on the axillary artery was also calculated to work out a margin of error that can be applied for the safe cannulation of the axillary artery.

The coracoacromial plane

**Figure 5:**
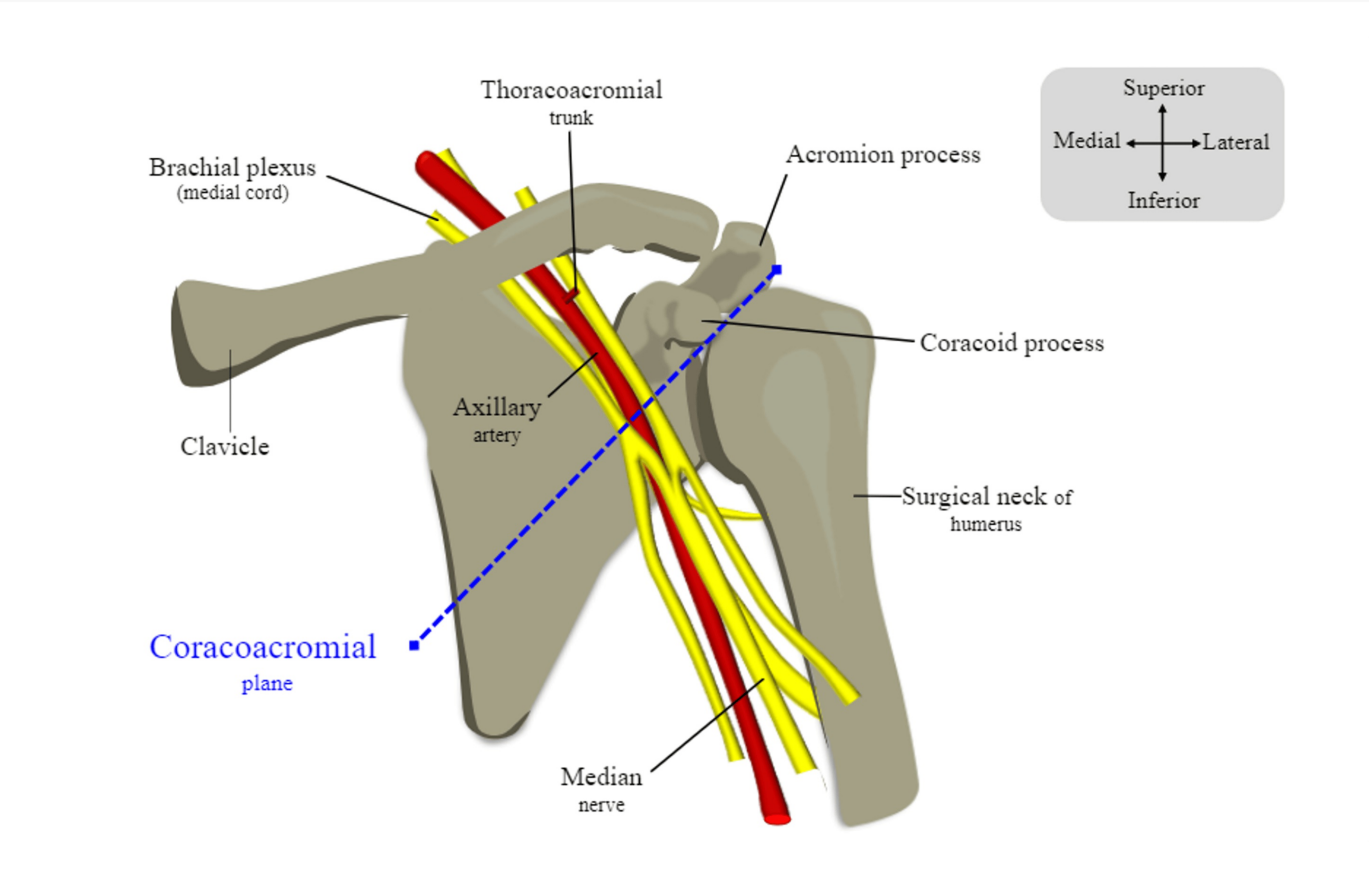
The coracoacromial plane. Diagram showing the coracoacromial plane that was used to carry out measurements of parameters 1-3. Image adapted from Mohammed et al. [6] with official permission and license granted to reproduce the image.

Figure 5 shows the plane that was used to carry out the measurements demarcated by the blue dashed line. To ensure reproducible results, the coracoid and the acromion were selected as bony landmarks. These landmarks are not only prominent and hence easy for reproducibility of results, but also transferable in the sense that they can be easily visualised in radiographs and other medical imaging. The plane extends from the anterolateral aspect of the acromion to the tip of the coracoid process. The plane is then extended to go past the axillary artery as can be seen in Figure 5. This plane was used to carry out measurements of parameters 1-3, which are explained in further detail below. This newly defined plane will be referred to as the coracoacromial plane in further sections of this project.

Parameter 1: acromion to coracoid process distance

**Figure 6:**
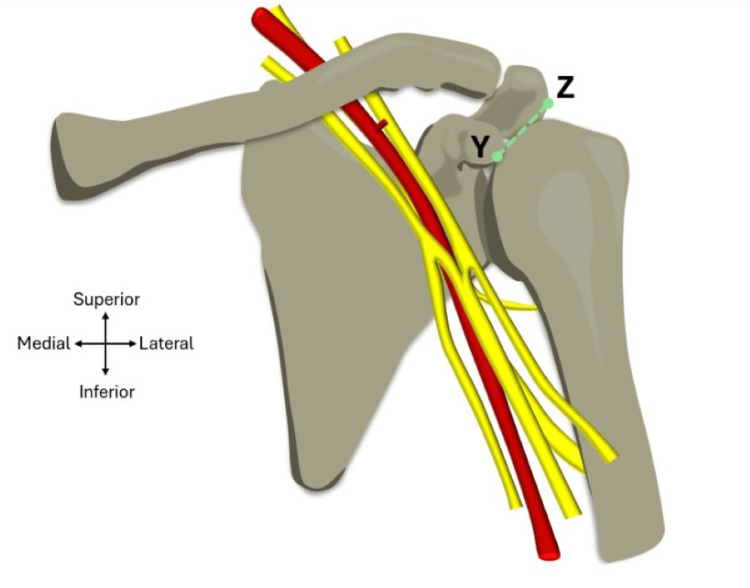
Parameter 1: acromion to coracoid process distance. Diagram showing the measurement taken from the anterolateral acromion (point Z) to the tip of the coracoid process (point Y) as represented by the green line. The measurement was taken at the level of the coracoacromial plane. Image adapted from Mohammed et al. [6] with official permission and license granted to reproduce the image.

The green line in Figure 6 shows a diagrammatic representation of the measurement taken between the acromion and the coracoid process. The lack of bony landmark features in the acromion process makes it challenging to carry out consistent measurements; therefore, point ‘Z’ was established as being lateral to the attachment of the coracoacromial ligament. The distance between point ‘Z’ to the tip of the coracoid process, which is labelled as point ‘Y’ in Figure 6, was taken to help quantify intra- and inter-observer error with regard to the measurement techniques, especially as these two bony landmarks have a static anterolateral relationship. This measurement was carried out using the string that was secured down by pins at the anterior acromial angle and the tip of the coracoid process. The use of a string allowed visualisation of the coracoacromial plane, which could be extended past the axillary artery and aided in later measurements (see parameter 2). Points on the string were recorded using surgical skin marker pens and the distance between the points was measured using an IP54 Faithfull Digital Calliper (Faithfull Tools, Norfolk, UK). The accuracy of these callipers is ±0.03 mm, with the resolution being 0.01 mm. To help mitigate observer bias, the digital display of the callipers was faced away to make sure it was not visible during the measuring process. Each measurement was carried out three times to calculate and record the mean result and standard deviation.

Parameter 2: diameter of axillary artery

**Figure 7:**
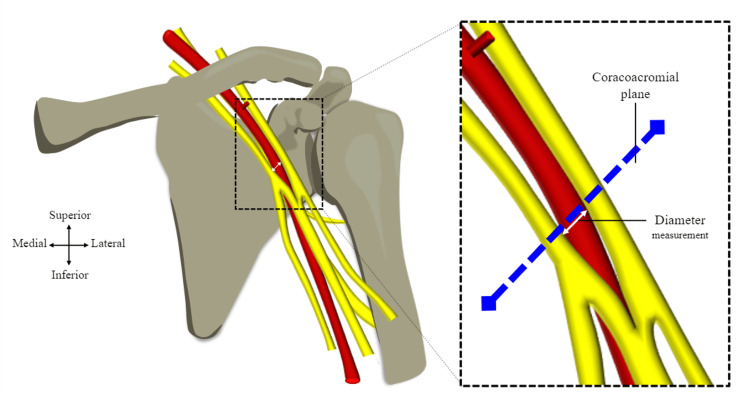
Parameter 2: diameter of axillary artery. Image showing the level at which the diameter was taken in the coracoacromial plane, which is a close-up of the area between the cords of the brachial plexus where the diameter was taken. Image adapted from Mohammed et al. [6] with official permission and license granted to reproduce the image.

The diameter of the axillary artery was taken at the level of the coracoacromial plane, as represented by the blue dashed line in Figure 7. The trajectory of the plane was maintained using a piece of string that was firmly secured with pins inserted into the acromion and tip of the coracoid process. The diameter was then taken at the inferior aspect of the plane, as shown in Figure 7. IP54 Faithfull Digital Callipers were positioned to ensure the digital display was not visible during the measuring process for the same reasons as described in parameter 1. Extra care was taken to ensure that whilst carrying out the measurement, the axillary artery was not compressed by the external measuring jaws of the callipers. Care was also taken to ensure that the true diameter of the vessel was taken by dissecting away any surrounding fat and fascia that could lead to an overestimation. It is also important to note that the diameter measurement in this study was not intraluminal, as the thickness of the arterial walls was included within the measurement. The measurement process was repeated three times and the mean result was calculated and recorded along with the standard deviation.

Parameter 3: coracoid to the safe point of the axillary artery distance

**Figure 8:**
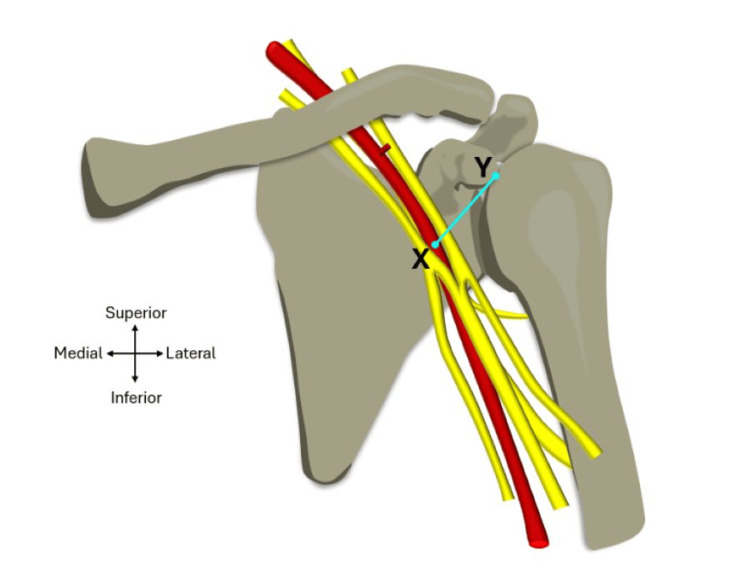
Parameter 3: coracoid to the safe point of the axillary artery distance. Diagram showing the points where the measurements for the distance between the coracoid process and the safe point on the axillary artery occurred. Point Y = tip of coracoid process; point X = safe point on the axillary artery. Image adapted from Mohammed et al. [6] with official permission and license granted to reproduce the image.

The safe point on the axillary artery is defined as the midpoint of the diameter of the axillary artery in the coracoacromial plane, represented as point X in Figure 8. The midpoint was calculated using the mean diameter (taken from the results of parameter 2) of each axillary artery and dividing it by two. The midpoint distance was then measured using the IP54 Faithfull Digital Callipers in the coracoacromial plane with the digital display facing away to reduce observer bias. A surgical marker pen was used to denote where the proposed ‘safe point’ on the axillary artery is located. The distance between the tip of the coracoid process (point Y) and the safe point on the artery (point X) was then measured along the length of the coracoacromial plane. This measurement was also taken three times for the mean result along with the standard deviation to be calculated and recorded.

Parameter 4: distance from the safe point to the median nerve

**Figure 9:**
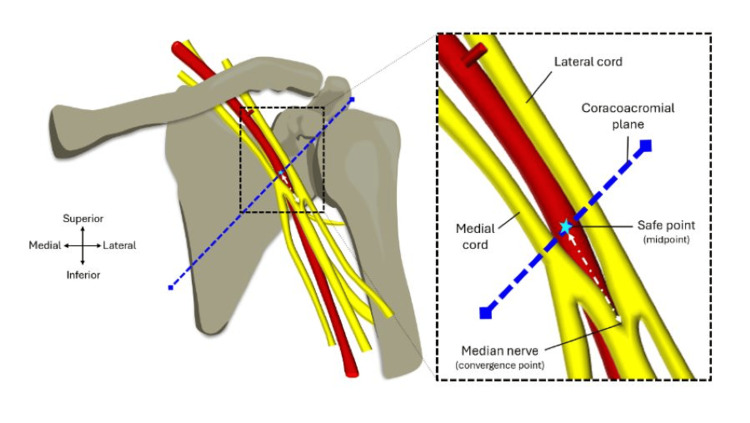
Parameter 4: distance from the safe point to the median nerve. Diagram showing the points where the measurements for the distance between the safe point on the axillary artery (indicated by the star) and the median nerve. Image adapted from Mohammed et al. [6] with official permission and license granted to reproduce the image.

The point where the medial and lateral cords of the brachial plexus converge anteriorly to the axillary artery demarcates the starting point of the median nerve and is depicted in Figure 9. A string was extended between the safe point on the axillary artery to the median nerve convergence point and two points were marked using a surgical pen. String was used as the measurement tool to follow the course of the vessel as this was not linear in nature. To help mitigate observer bias, the distance between the two points of the string was measured using the IP54 Faithfull Digital Callipers making sure that the digital display was not visible. A new section of string was used each time. This measurement was repeated three times to calculate and record the mean length and standard deviation.

Parameter 5: distance from the safe point and the thoracoacromial trunk

**Figure 10:**
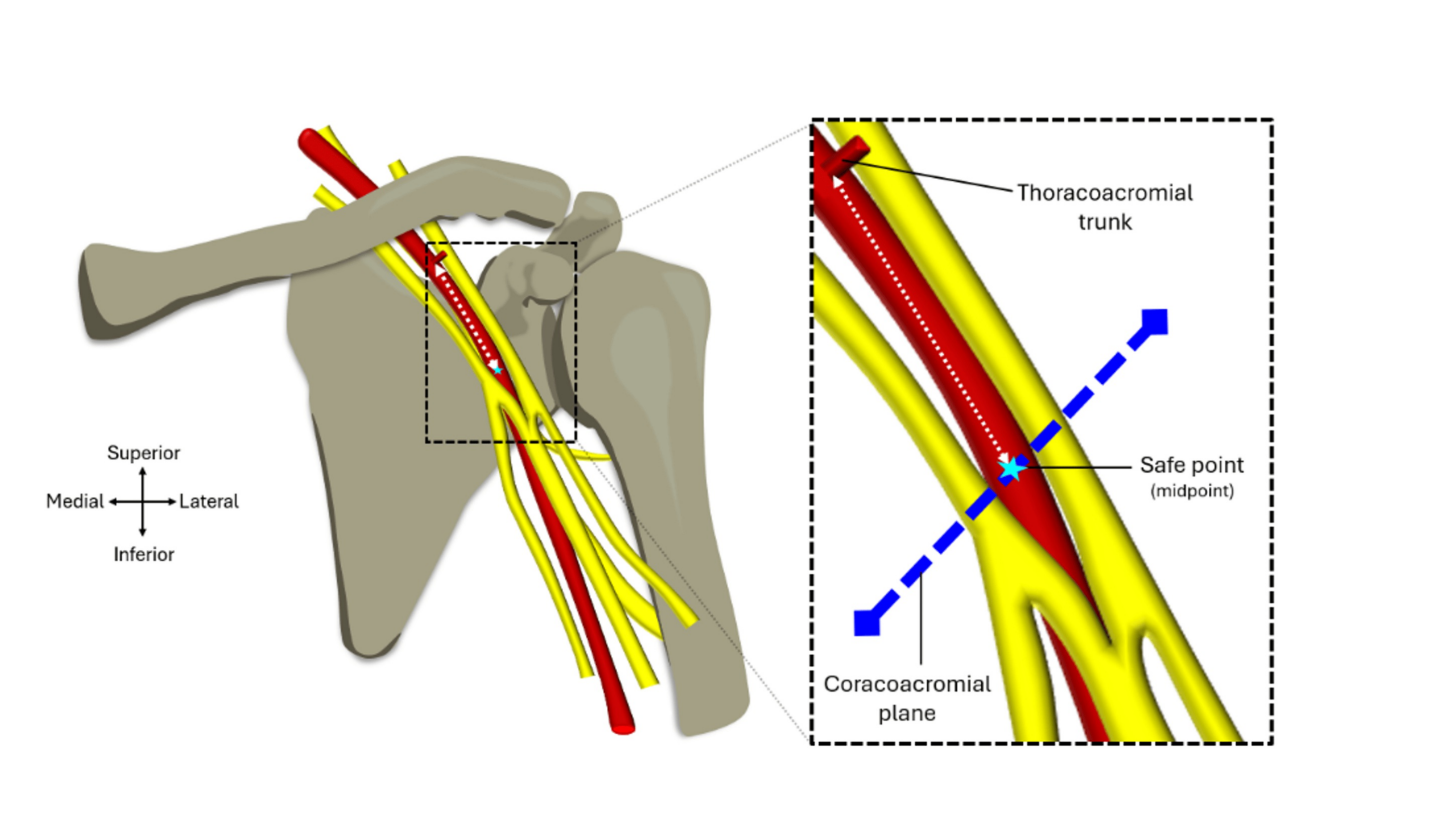
Parameter 5: distance from the safe point and the thoracoacromial trunk. Diagram showing the left anterior shoulder region with the medial and lateral cords of the brachial plexus being visible along with the nerves of the brachial plexus. Image taken from Mohammed et al. [6] and adapted to include the thoracoacromial trunk with official permission and license granted to reproduce the image.

The natural trajectory of the axillary artery could be followed using string to measure the length from the safe point of the axillary artery (determined via parameter 3) and the inferior margin of the thoracoacromial trunk, which is represented by the white dotted line in Figure 10. Using a surgical marker pen, both the safe point and distal base of the thoracoacromial trunk were marked on a piece of string. The distance between these two points was then measured with the digital display of the IP54 Faithfull Digital Callipers facing away to mitigate observer bias. The measurement was repeated three times and a mean length along with the standard deviation was calculated and recorded.

Area of safety

**Figure 11:**
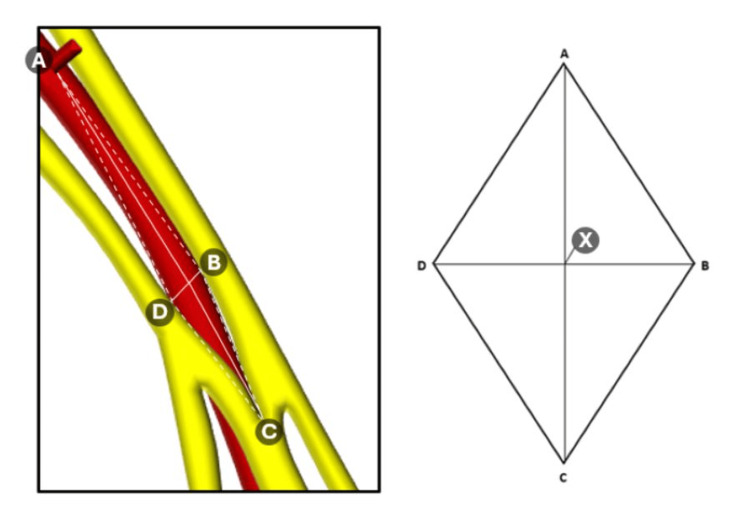
Area of safety. Diagram outlining the area of safety created by four landmark points based on the following measurements: A-X = safe point to the thoracoacromial trunk; C-X = safe point to convergence point of the median nerve; B-D = diameter of axillary artery at the coracoacromial plane. Point X represents the midpoint (or 'safe point') of the axillary artery.

The aim of this project was to test whether the plane between the acromion and coracoid process could be used to find an area of safety on the axillary artery to gain vascular access with minimal risk to the brachial plexus or branches of the thoracoacromial trunk. Measurements 2, 4, and 5 allow for the calculation of an area of safety to be worked out around a defined safe point on the axillary artery, which is labelled in Figure 11 as point X. Point A represents the thoracoacromial trunk, point B represents the medial margin of axillary artery, point C represents the origin of the median nerve, and point D represents the lateral edge of the axillary artery. Collectively these points form a diamond shape that is known mathematically as a rhombus. Using the triangulation of these anatomical landmarks allows for a margin of error to be calculated and compared.

The area of a rhomboid can be calculated using the equation described in the results section, which involves adding the lengths of parameters 4 and 5, multiplying this with parameter 2, and then dividing the answer by two. The mean lengths for each individual were used for this calculation.

Statistical analysis

A paired two-tailed Student's t-test was selected to determine the significance of variation between the data that were collected. The tests were carried out using Microsoft Excel with a data analysis add-on used for the statistical calculations. A p-value of less than 0.05 was considered significant for this study. The results from cadaver 1 were not included in any of the statistical calculations since only the data from one (left) side of the cadaver were available. The results of the statistical analysis are detailed in the table of results for each respective measurement.

## Results

The data collected from each cadaver have been recorded in various tables that are presented in this section alongside results from the statistical analysis. A summary of key findings has also been provided at the end of this section.

Parameter 1: distance between the acromion and the coracoid process

Table 2 outlines the measurements taken between the acromion and coracoid process from the nine deceased donors available for the study. No right-sided measurements were recorded for cadaver 1 since it is used for teaching purposes at Keele Medical School; hence those measurements have been denoted as not applicable (n/a) in the table. A total of three measurements were taken for each side, results for which have been shown below in columns named 1st, 2nd, and 3rd measurements. A mean for those results along with the standard deviation for both sides of each cadaver was also calculated and can be seen in the final two columns on the right side of the table. The standard deviation for each dataset was less than ±1 mm for each cadaver, suggesting that the measurement technique was consistently and reliably implemented. Focusing on the data for cadavers 2 to 9, the average distance from the acromion to the coracoid process was similar on both the left and right sides of the cadaver, thus suggesting there is no significant variation between the two sides. This was confirmed by carrying out a Student’s t-test paired for means, which generated a p-value of 0.081. Since p ≥ 0.05, the t-test confirms there is no significant difference between the right and left mean measurements; therefore, increasing the confidence in using the acromion process and coracoid process as bony landmarks to aid in axillary artery cannulation bilaterally. Furthermore, since both the acromion and the coracoid process are features of one bone, any movement of the upper limb will cause these parts of the scapula to move relative to one another, making them not only palpable but also stable landmarks.

**Table 2:** Parameter 1: distance between the acromion and the coracoid process. The distance between the acromion and the coracoid process was measured in millimetres (mm) on both the left and right sides of the cadavers. n/a: not applicable.

	Left measurements (mm)			Right measurements (mm)				
Cadaver	1^st^	2^nd^	3^rd^	1^st^	2^nd^	3^rd^	Left, mean ± standard deviation (mm)	Right, mean ± standard deviation (mm)
1	62.08	63.14	62.37	n/a	n/a	n/a	62.53 ± 0.45	n/a
2	40.02	41.81	40.96	42.62	41.93	42.80	40.93 ± 0.73	42.45 ± 0.37
3	45.33	46.14	45.93	47.41	48.91	47.91	45.80 ± 0.34	48.08 ± 0.62
4	46.89	47.13	47.03	50.26	51.31	49.34	47.02 ± 0.10	50.30 ± 0.80
5	50.64	51.08	50.17	52.91	53.06	52.13	50.63 ± 0.37	52.70 ± 0.41
6	47.65	48.01	47.95	47.05	47.81	47.13	47.87 ± 0.16	47.33 ± 0.34
7	46.07	45.99	45.76	43.31	42.96	42.99	45.94 ± 0.13	43.09 ± 0.16
8	50.46	49.85	50.22	57.09	56.88	56.59	50.18 ± 0.25	56.85 ± 0.20
9	33.38	34.01	33.72	37.98	38.04	37.83	33.70 ± 0.26	37.95 ± 0.09
Total means							47.18	47.34

Parameter 2: diameter of the axillary artery in the coracoacromial plane

The second measurement relies on the coracoacromial plane to measure the diameter of the axillary artery, with the data and results presented in Table 3. The midpoint of the diameter in this plane was subsequently used to define the ‘safe point’ of cannulation in each artery, as explained in the methods section.

**Table 3:** Parameter 2: diameter of the axillary artery in the coracoacromial plane. The diameter of the axillary artery at the coracoacromial plane was measured in millimetres (mm) on both the left and right sides of the cadavers. n/a: not applicable.

	Left measurements (mm)			Right measurements (mm)				
Cadaver	1^st^	2^nd^	3^rd^	1^st^	2^nd^	3^rd^	Left, mean ± standard deviation (mm)	Right, mean ± standard deviation (mm)
1	8.87	8.32	8.96	n/a	n/a	n/a	8.72 ± 0.28	n/a
2	9.11	9.03	9.21	8.96	8.29	8.44	9.12 ± 0.07	8.56 ± 0.29
3	7.91	7.88	8.03	8.32	8.53	7.93	7.94 ± 0.06	8.26 ± 0.25
4	9.14	9.26	8.98	8.72	8.14	7.96	9.13 ± 0.11	8.27 ± 0.32
5	10.79	10.62	11.01	10.42	10.17	10.91	10.81 ± 0.16	10.50 ± 0.31
6	8.92	8.56	8.33	9.40	9.13	9.70	8.60 ± 0.24	9.41 ± 0.23
7	9.89	10.13	9.56	8.77	8.32	8.91	9.86 ± 0.23	8.66 ± 0.25
8	9.30	9.61	9.15	9.13	9.29	9.86	9.35 ± 0.19	9.43 ± 0.31
9	7.75	7.92	7.81	7.24	8.01	7.31	7.82 ± 0.07	7.52 ± 0.35
Total means							9.04	8.83

From the total mean measurements of the vessel diameter across all the cadavers used in the study, the results suggest there are no significant differences in the sizes between the two sides, with the left side averaging 9.04 mm and the right side being 8.83 mm. Some may expect the diameter of the axillary artery would be wider on the left compared to the right due to the different branching patterns, as detailed in the introduction. Whilst this concept is true of the total means, the diameter of the right axillary artery is not consistently narrower compared to the left side. To confirm whether there were any significant differences between the two sides, the Student's t-test was carried out and resulted in p = 0.31. Although p ≥ 0.05, which confirms there is no significant difference between the two sides of the body in regard to the diameter of the artery, these results should be interpreted in the context of embalmed individuals. At the time of this project being carried out, some of the deceased donors were stored in a fixed position for two years, which may have differed slightly across each individual and may have influenced the ease of taking the diameter measurements at the coracoacromial plane.

Parameter 3: distance between the coracoid process and the safe point of the axillary artery

The third measurement was taken from the tip of the coracoid process to the safe point of the axillary artery (as calculated by the midpoint of the mean diameter of the artery in the coracoacromial plane), with the data displayed in Table 4.

**Table 4:** Parameter 3: distance between the coracoid process and the safe point of the axillary artery. The distance of the coracoid to the safe point of the axillary artery was measured in millimetres (mm) on both the left and right sides. n/a: not applicable.

	Left measurements			Right measurements				
Cadaver	1^st^	2^nd^	3^rd^	1^st^	2^nd^	3^rd^	Left, mean ± standard deviation	Right, mean ± standard deviation
1	31.79	32.13	31.55	n/a	n/a	n/a	31.82 ± 0.24	n/a
2	41.21	40.98	41.33	38.33	39.16	39.52	41.17 ± 0.15	39.00 ± 0.50
3	37.60	38.14	37.58	43.18	43.93	42.18	37.77 ± 0.26	43.10 ± 0.72
4	27.28	28.11	28.03	35.67	34.18	35.93	27.81 ± 0.37	35.26 ± 0.77
5	36.41	37.08	36.93	34.37	34.11	35.51	36.81 ± 0.29	34.66 ± 0.61
6	52.79	52.14	51.91	48.59	47.93	48.01	52.28 ± 0.37	48.18 ± 0.29
7	35.56	35.61	35.03	34.08	34.19	35.16	35.40 ± 0.26	34.48 ± 0.49
8	25.17	25.41	26.08	38.45	38.51	39.16	25.55 ± 0.39	38.71 ± 0.32
9	36.05	36.15	36.27	36.00	36.81	37.13	36.16 ± 0.09	36.65 ± 0.48
Total means							36.12	38.76

As evidenced from the raw measurements, a range of results was obtained for the length between the midpoint of the axillary artery in the coracoacromial plane to the coracoid process; the smallest distance on the left was 27.81 mm and the largest being 52.28 mm. This ±24.5 mm range made it difficult to form a solid relationship between the bony landmarks used in the project with the targeted vessel. The total mean results gathered from the dataset show that between the left and right sides, there was a difference of approximately 2 mm. The Student's t-test carried out for the data resulted in p = 0.34, which means that p ≥ 0.05 indicates no significant difference between the two sides.

To determine whether a relationship between parameter 3 (coracoid to the safe point of the axillary artery distance) and parameter 1 (distance between the acromion and the coracoid process) was present, the ratios between the mean measurements were worked out for both sides and can be seen in Tables 5, 6.

**Table 5:** Ratio of measurements on the left side. The ratio of average measurements on the left side. Distance from the acromion to the coracoid process (parameter 1) compared to the length between the coracoid process and the defined ‘safe point’ on the axillary artery (parameter 3).

Cadaver	Mean of parameter 1 (mm)	Mean of parameter 3 (mm)	Left side ratio
1	62.53	31.82	1.97:1.00
2	40.93	41.17	0.99:1.00
3	45.80	37.77	1.21:1.00
4	47.02	27.81	1.69:1.00
5	50.63	36.81	1.38:1.00
6	47.87	52.28	0.91:1:00
7	45.94	35.40	1.29:1.00
8	50.18	25.55	1.96:1.00
9	33.7	36.16	0.93:1.00

**Table 6:** Ratio of measurements on the right side. The ratio of average measurements on the right side. Distance from the acromion to the coracoid process (parameter 1) compared to the length between the coracoid process and the defined ‘safe point’ on the axillary artery (parameter 3). N/A: not applicable.

Cadaver	Mean of parameter 1 (mm)	Mean of parameter 3 (mm)	Right side ratio
1	N/A	N/A	N/A
2	42.45	39.00	1.09:1.00
3	48.08	43.10	1.12:1.00
4	50.30	35.26	1.43:1.00
5	52.70	34.66	1.52:1.00
6	47.33	48.18	0.98:1.00
7	43.09	34.48	1.25:1.00
8	56.85	38.71	1.47:1.00
9	37.95	36.65	1.04:1.00

Table 5 shows the ratio of measurements for the left side. Cadavers 1 and 8 show that there is roughly a 2:1 ratio, meaning that the distance between the acromion process and the coracoid process is roughly twice that of the distance between the coracoid process and the safe point on the axillary artery. The ratio calculations of cadavers 2, 6, and 9 show there is roughly a 1:1 ratio, meaning that the distance between the acromion and the coracoid process is roughly the same as the distance between the coracoid and the safe point on the axillary artery.

Table 6 shows the ratio of measurements for the ride side. Note that due to cadaver 1 being used for teaching purposes, the right axillary artery was not dissected; hence the ratio of measurements has been denoted as N/A.

Although this is a small sample size, the results show that cadavers 2, 6, and 9 had roughly a 1:1 ratio, which was a pattern reflected from the left side that supports the findings that the distance between the bony landmarks of the scapula is approximately the same as the distance between the coracoid process and the safe point on the axillary artery. The results for cadavers 4, 5, and 8, however, showed an approximate ratio of 1.5:1, indicating that the distance between the acromion and the coracoid process is roughly 1.5 times greater than the distance between the coracoid process and the safe point on the axillary artery. Cadavers 2 and 7 had no clear relationships of note.

Although a few of the cadavers had a similar ratio relationship, the range varied hugely; for example, between 1:1 to 2:1 on the left side and 1:1 to 1:1.5 on the right side, which clinically is not useful. This was confirmed by carrying out a Pearson’s correlation test.

The critical value for Pearson’s correlation coefficient (r) for 7 degrees of freedom at a significance level of 0.05 was 0.66. The coefficient value obtained showed a slight negative correlation with -0.24, which is lower than 0.66 and indicates no significant correlation.

The critical value for Pearson’s correlation coefficient (r) for 6 degrees of freedom at a significance level of 0.05 was 0.71. The coefficient value obtained from this dataset was 0.035 (slight positive correlation), which is lower than 0.71 and also indicates there is no significant correlation.

**Figure 12:**
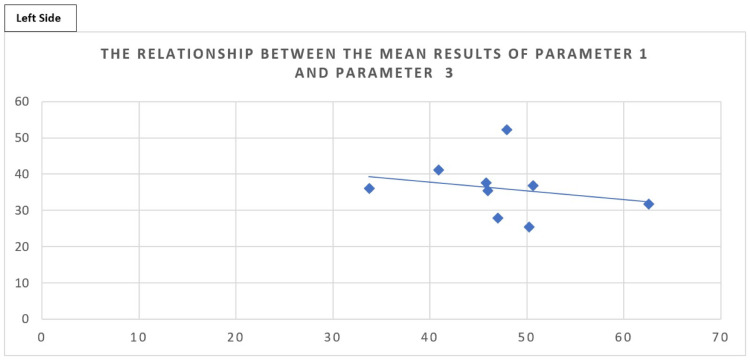
Scatter graph showing the relationship between parameter 1 (x-axis) and parameter 3 (y-axis) for the left side. Scatter graph showing the relationship between parameter 1 (x-axis), the mean distance between the acromion and the coracoid process, and parameter 3 (y-axis), the mean distance between the coracoid process and the safe point of the axillary artery, with data being clustered around one area without a clear relationship. The trend line shows a weak negative correlation between the two variables. These are the data for the left side.

**Figure 13:**
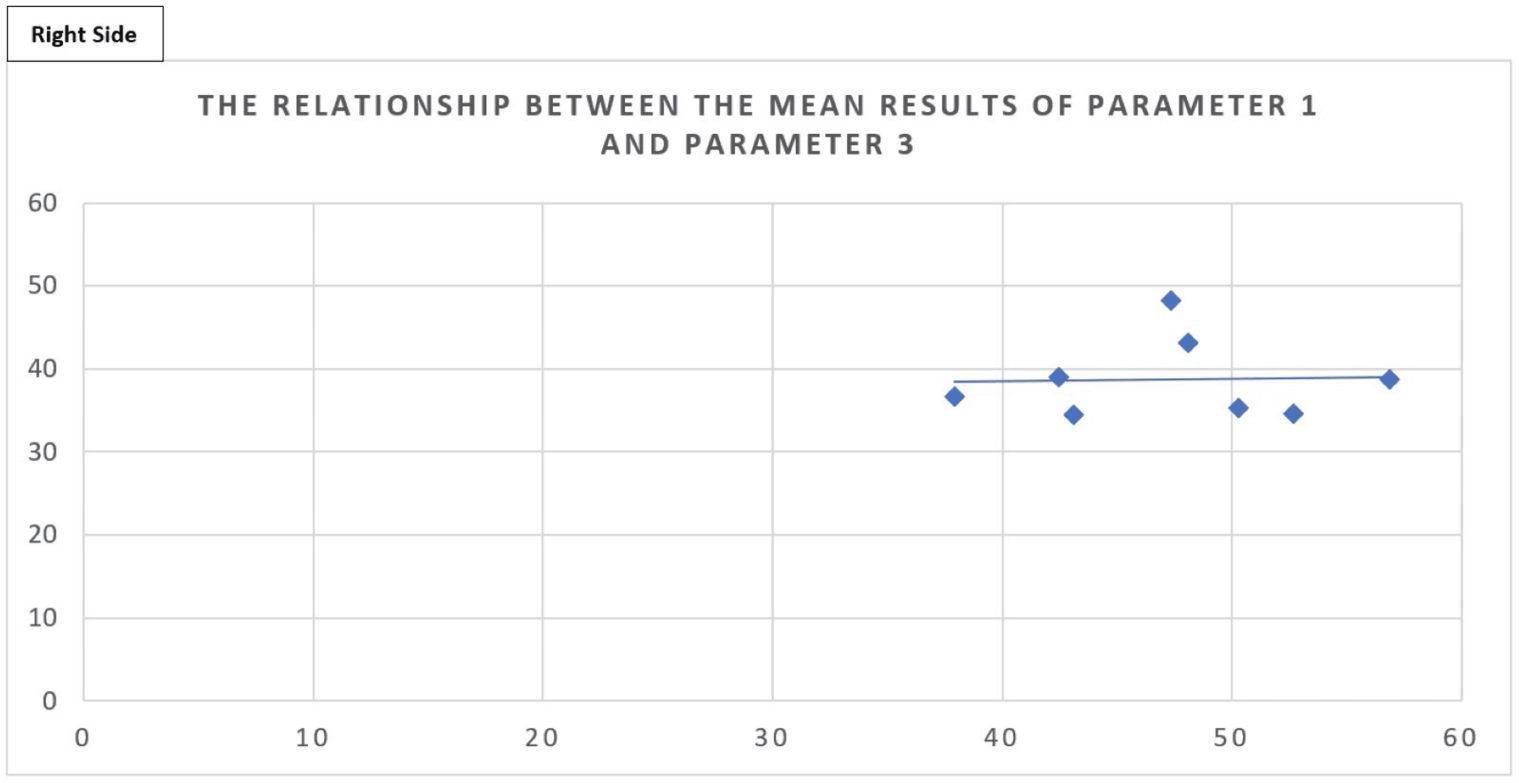
Scatter graph showing the relationship between parameter 1 (x-axis) and parameter 3 (y-axis) for the right side. Scatter graph showing the relationship between parameter 1 (x-axis), the mean distance between the acromion and the coracoid process, and parameter 3 (y-axis), the mean distance between the coracoid process and the safe point on the axillary artery, with data being clustered around one area without a clear relationship. The trend line shows a very weak positive correlation between the two variables. These are the data for the right side.

The mean datasets from parameters 1 and 3 have also been plotted on scatter graphs with Figures 12, 13, highlighting a lack of correlation between the distance from the acromion to the coracoid and the length from the coracoid to the axillary artery safe point for the left and the right sides, respectively.

Figure 12 shows a weakly negative correlation between parameter 1 and parameter 3 on the left side as confirmed by Pearson's correlation carried out and for the right side. There was a very weakly positive correlation that can be seen in Figure 13; however, both are non-significant as confirmed by the Pearson's correlation test.

Parameter 4: distance between the safe point and the median nerve

Table 7 below contains the data from parameter 4, which was taken between the safe point on the axillary artery and the median nerve of the brachial plexus.

**Table 7:** Parameter 4: distance between the safe point on the axillary artery and the median nerve measured in millimetres (mm) on both the left and right sides of the cadaver. n/a: not applicable.

	Left measurements (mm)			Right measurements (mm)				
Cadaver	1^st^	2^nd^	3^rd^	1^st^	2^nd^	3^rd^	Left, mean ± standard deviation	Right, mean ± standard deviation
1	18.84	18.52	19.01	n/a	n/a	n/a	18.79 ± 0.20	n/a
2	10.56	10.11	10.64	8.43	8.22	8.91	10.44 ± 0.23	8.52 ± 0.29
3	19.01	19.28	19.14	29.08	29.13	29.44	19.14 ± 0.11	29.22 ± 0.16
4	4.72	4.18	4.31	4.25	3.96	4.18	4.40 ± 0.23	4.13 ± 0.12
5	29.35	29.08	29.17	76.17	76.99	75.85	29.20 ± 0.11	76.34 ± 0.48
6	17.92	18.03	17.83	35.77	34.93	35.50	17.93 ± 0.08	35.40 ± 0.35
7	70.81	70.11	70.60	40.29	41.16	40.99	70.51 ± 0.29	40.81 ± 0.38
8	9.76	9.52	9.81	7.31	7.98	7.83	9.70 ± 0.13	7.71 ± 0.29
9	29.25	29.06	29.33	14.76	14.17	15.02	29.21 ± 0.11	14.65 ± 0.36
Total means							23.25	27.10

Measurements from both the left and right sides of the cadaver can be seen in the table above with the mean measurements seen in the final two columns. From the measurements, it is clear to see that there were vast differences in the measurements across the different cadavers. Two cadavers in particular (cadaver 5 - right side, cadaver 7 - left side) have vastly different sets of measurements compared to others. This was mostly due to haematoma formation and lymph node enlargement distorting the anatomy around the brachial plexus and artery giving rise to particularly larger measurements. Smaller differences in the measurements may be due to inconsistent dissection of the axillary sheath and handling of the brachial plexus during teaching sessions by 1st and 2nd-year medical students at Keele University, which could have led to disruption in the typical position of the anatomy. It is important to note that the position of the median nerve in relation to the safe point was always distal. This was true for all of the cadavers that were included in this study both on the left and right sides. The safe point of the axillary artery in the coracoacromial plane was consistently located in the 2nd part of the axillary artery and the formation of the median nerve occurred at the 3rd part of the axillary artery.

The total mean of the data was also worked out and can be seen on the bottom row of Table 7, with the means only varying by approximately 4 mm between the left and the right side. The Student's t-test was carried out and resulted in p = 0.70, which is greater than 0.05, indicating that the difference between the measurements on the left versus right was not significant.

Parameter 5: distance from the safe point to the thoracoacromial trunk

The distance from the safe point and the thoracoacromial trunk was measured and Table 8 below contains the results for this.

**Table 8:** Parameter 5: distance from the safe point and the thoracoacromial trunk measured in millimetres (mm) on both the left and right sides of the cadavers. n/a: not applicable.

	Left measurements (mm)			Right measurements (mm)				
Cadaver	1^st^	2^nd^	3^rd^	1^st^	2^nd^	3^rd^	Left, mean ± standard deviation	Right, mean ± standard deviation
1	12.40	13.21	12.89	n/a	n/a	n/a	12.83 ± 0.33	n/a
2	9.96	9.21	9.59	8.33	8.16	8.31	9.58 ± 0.31	8.26 ± 0.08
3	5.65	5.81	5.71	12.58	12.41	12.03	5.72 ± 0.07	12.34 ± 0.23
4	7.22	7.13	7.08	14.02	14.15	14.73	7.14 ± 0.06	14.30 ± 0.31
5	17.98	17.70	17.57	20.34	20.18	20.71	17.75 ± 0.17	20.41 ± 0.22
6	2.86	2.93	2.71	1.23	1.11	1.03	2.83 ± 0.09	1.12 ± 0.08
7	14.96	15.34	14.50	18.88	19.16	18.53	14.93 ± 0.34	18.86 ± 0.26
8	6.69	6.29	6.44	11.97	11.83	11.29	6.47 ± 0.16	11.70 ± 0.29
9	24.46	24.01	25.06	18.52	18.91	18.71	24.51 ± 0.43	18.71 ± 0.16
Total means							11.31	13.21

Measurements for both the right and left side for parameter 5 were taken three times and the mean was worked out, which can be seen in the final two columns. The total mean of the averages was also calculated and presented in the final row. Measurements varied hugely between the cadavers indicating great variation in where this branch of the axillary artery originated from. The thoracoacromial trunk measurements for cadaver 6 are indicative of the smallest distance away from the coracoacromial plane and in this small sample size act as an outlier. This was most likely due to this cadaver being embalmed whilst the shoulders were slightly elevated on both sides, which may have affected the distance measurements for this particular case.

The safe point on the axillary artery was situated in the 2nd part of the axillary artery, which is the same part of the artery where the thoracoacromial trunk originates. Despite the relatively close proximity, the coracoacromial plane located the safe point of the axillary artery such that the thoracoacromial trunk was bilaterally always proximal on each of the cadavers. The Student's t-test generated a p-value of 0.24, which is greater than 0.05, hence showing no significant difference between this distance on the left versus the right side of the cadaver.

Area of safety calculations

**Figure 14:**
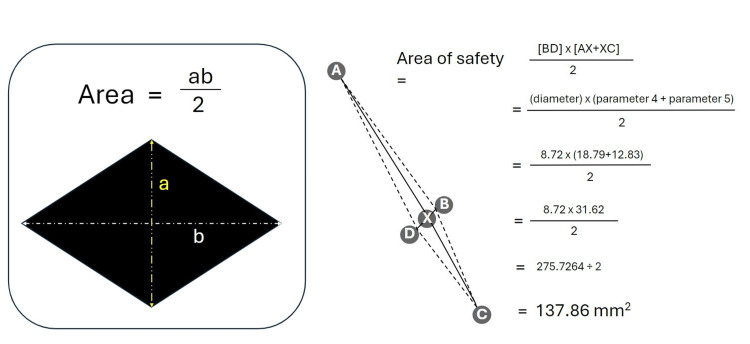
Calculating the area of a rhombus. The area of safety is created by four landmark points with A = thoracoacromial trunk, B = medial edge of axillary artery, C = median nerve, and D = lateral edge of axillary artery. Point X represents the safe point on the axillary artery as defined by the coracoacromial plane. The figure shows a worked example of how the area of safety was calculated using the results of the left side for cadaver 1.

The area of safety was calculated using the formula for the area of a rhombus: area = ab/2. This formula was selected since the points of measurement correspond to the shape of a rhombus, as seen in Figure 14.

All the calculations used the mean distances with the details summarised in Table 9 below containing the results of this calculation for all the other cadavers for both the right and left sides.

**Table 9:** The area of safety calculations for both the right and left sides of the cadavers. The measured areas of safety for each cadaver on the left and right side using the results for the mean diameter of the axillary artery and the distance of the safe point from the thoracoacromial trunk (parameter 4) and median nerve (parameter 5). N/A: not applicable.

Left					Right				
Cadaver	Mean of diameter (mm) (BD)	Mean of parameter 4 (mm) (XC)	Mean of parameter 5 (mm) (AX)	Area of safety (mm^2^)	Cadaver	Mean of diameter (mm) (BD)	Mean of parameter 4 (mm) (XC)	Mean of parameter 5 (mm) (AX)	Area of safety (mm^2^)
1	8.72	18.79	12.83	137.86	1	N/A	N/A	N/A	N/A
2	9.12	10.44	9.58	91.29	2	8.56	8.52	8.26	71.82
3	7.94	19.14	5.72	98.69	3	8.26	29.22	12.34	171.64
4	9.13	4.40	7.14	52.68	4	8.27	4.13	14.30	76.21
5	10.81	29.20	17.75	253.76	5	10.50	76.34	20.41	507.94
6	8.60	17.93	2.83	89.27	6	9.41	35.40	1.12	171.83
7	9.86	70.51	14.93	421.22	7	8.66	40.81	18.86	258.37
8	9.35	9.70	6.47	75.59	8	9.43	7.71	11.70	91.52
9	7.82	29.21	24.51	210.05	9	7.52	14.65	18.71	125.43
Total mean				158.93	Total mean				184.37

The mean area of safety on the left side was 158.93 mm^2^ compared to the right side, which was 184.37 mm^2^, indicating that the area of safety was larger on the right side. However, to identify any significant differences between the two sides, the Student's t-test was carried out and resulted in a p-value of 0.62, which is greater than 0.05, confirming no significant differences exist between the two sides.

## Discussion

The aim of this project was to investigate close anatomical relationships of the axillary artery and establish whether palpable bony landmarks could help identify a ‘safe’ area for cannulation whilst reducing the risk of complications often associated with a transaxillary approach. The results from this initial research indicate the distance between the acromion and coracoid process has no clear relationship to the distance between the coracoid process and the proposed safe point on the axillary artery. This was supported by the ratio calculations and results from Pearson’s correlation statistical test that determined no correlation for either the left or right side of the body.

There is very little literature looking at bony landmarks and their distance from the axillary artery; however, a study conducted in 2004 by Lo et al. [7] was carried out on five fresh-frozen cadaveric shoulders and looked at the distance from the coracoid process to adjacent structures, including the axillary artery. In the study, the measurements were carried out from the tip of the coracoid process (similar to this project) and precision callipers were used to record the ‘minimal’ distance to the axillary artery. The mean result obtained was 36.8 ± 6.1 mm; however, this was a mean of the overall distances and was not categorised into separate left and right sides. The data generated from this project found that the mean distance between the coracoid and axillary artery was 36.12 mm on the left and 38.76 mm on the right. These distances are very similar to the article by Lo et al. [7], which could stem from the fact that the methodology used for the measurements was very similar. Whilst the results are similar, it is important to note that Lo et al. [7] determined the ‘minimal’ distance and this may not have necessarily been consistent across each cadaver; for example, the minimal length in one individual may have spanned from the tip of the coracoid to the 2nd part of the axillary artery whereas in another cadaver the shortest distance may have been to the 1st part of the axillary artery instead. However, having said that, getting measurements that were similar to research already published elsewhere increases confidence in the results obtained in this project.

A recent study by Stone et al. [8] also measured the distance between the tip of the coracoid process and the axillary artery diagonally in a sample of nine fresh whole-body cadavers. Measurements were carried out using fluoroscopy and the results obtained showed a mean distance of 46.5 mm from the coracoid to the axillary artery. This result differed by approximately 10 mm compared to the results from this project and the study by Lo et al. [7], which may be explained by the methodological approach since Stone et al. [8] used fluoroscopic measurements.

**Figure 15:**
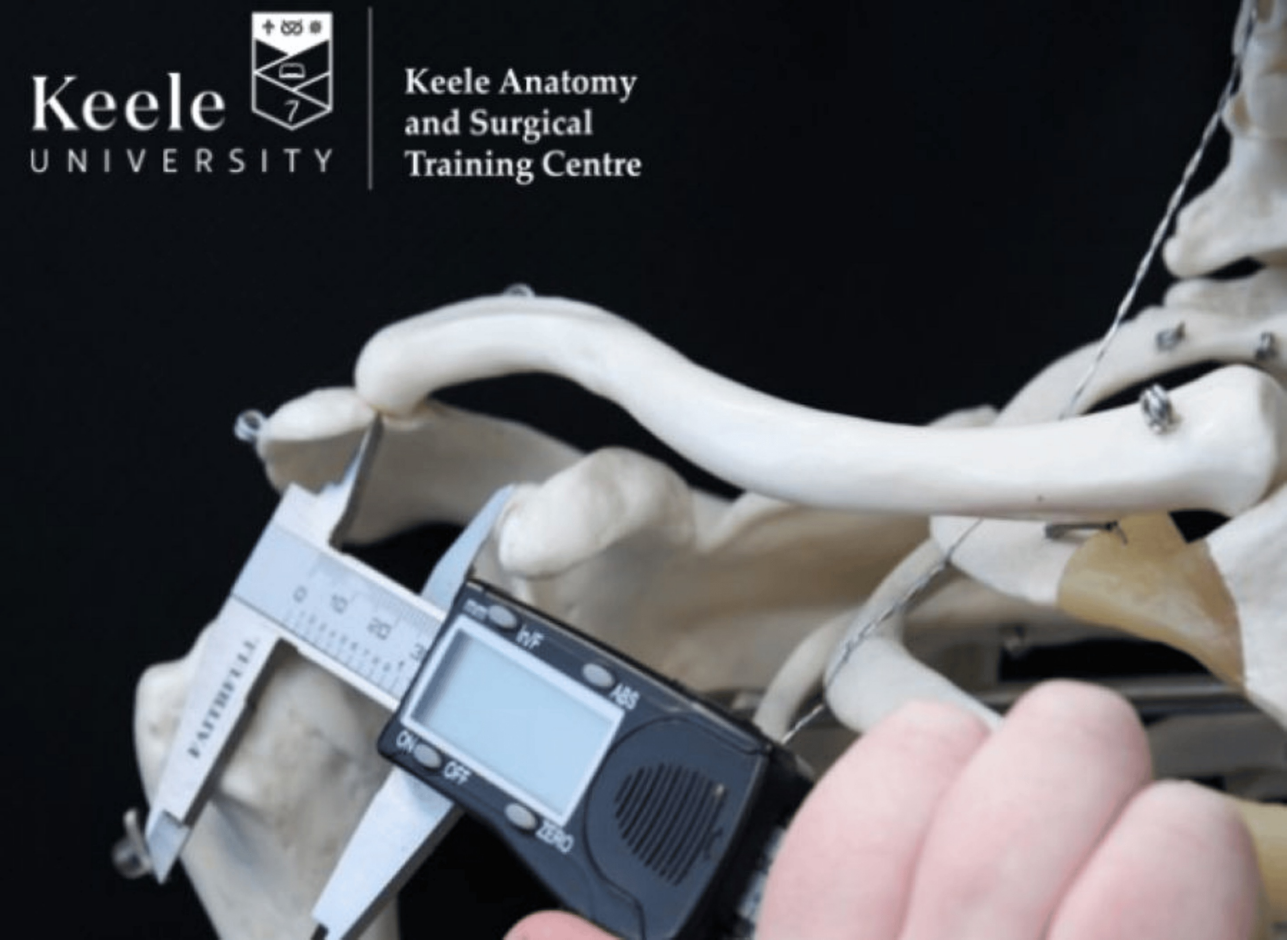
Measurement of the coracoacromial distance.

Measurements between the acromion process and the coracoid process have not been vastly carried out in research, perhaps due to a lack of significance for this measurement previously. One study conducted by Saha and Vasudeva [9] focused on the distance between the acromion and the coracoid process as part of a project looking to carry out a morphometric evaluation of the acromion. The results of that study showed a mean distance of 28.34 mm in a sample of 200 dry adult scapulae. Whilst this is vastly different from the results obtained from this project, it is vital to note that the measurements by Saha and Vasudeva [9] were taken in a similar fashion to what has been replicated in Figure 15. The figure shows the callipers positioned between the medial border of the acromion and the lateral border of the coracoid process, whereas, in this project, the distance was actually measured between the anterior lateral aspect of the acromion and the tip of the coracoid process due to keeping the scapular ligaments and other soft tissue structures intact. This may account for the approximately 20 mm difference between the results from this project and from Saha and Vasudeva [9].

Although there was no particular relationship between the acromion and the coracoid, the coracoacromial plane that was created using these bony landmarks was helpful since the trajectory traversed the 2nd part of the axillary artery and in a section where it was consistently distal to the thoracoacromial trunk as well as being consistently proximal to the formation of the median nerve. Therefore, it can be concluded that cannulation of the axillary artery in the coracoacromial plane made up using the acromion and the coracoid process will allow the cannula to be inserted into a safe area of the artery.

This plane consistently located an area where there were no cords of the brachial plexus traversing the anterior aspect of the artery, whereby the convergence of the lateral and medial cords to form the median nerve would always be below the point of cannulation. The thoracoacromial branch of the axillary artery would also always be above the point of cannulation in the coracoacromial plane, and this was the case for each cadaver that was present in this study. This is evident from the data collected for parameters 4 (distance away from the median nerve) and 5 (distance away from the thoracoacromial trunk) in the results section. The data showed that on average, on the left side, the mean distance between the safe point on the axillary artery and the median nerve below for all the cadavers was 23.25 mm on the left and 27.10 mm on the right side, which indicates that the median nerve was below the safe point of axillary cannulation in both left and right sides of every cadaver. The Student's t-test carried out on this also showed the p-value being 0.70, which is greater than 0.05, indicating no significant variation between the left and right sides. Furthermore, the results for the mean distance between the safe point on the axillary artery and the thoracoacromial trunk were calculated as being 11.31 mm on the left and 13.21 mm on the right. The Student's t-test carried out on this set of data resulted in a p-value of 0.24, which is greater than 0.05, indicating no significant variation between the left and right sides of the body. This therefore suggests that the cannulation in the coracoacromial plane made up using the coracoid process and the acromion enables cannulation to take place in a safe manner on both sides of the body making the coracoid process and the acromion suitable bony landmarks for this procedure.

There is very little research that has been carried out on distances between the median nerve and the thoracoacromial trunk of the axillary artery; however, typical anatomy seen in this region is that the thoracoacromial trunk arises at the proximal part of the 2nd part of the axillary artery and the median nerve forms as the medial and the lateral cords converge around the 3rd part of the axillary artery with the majority of the 2nd part of the axillary artery having no nerves that traverse the anterior section of the artery at this level also [10]. However, there are also various variations that can be seen in the branching patterns from the axillary artery [11] and in the formation of the brachial plexus, which have been extensively reported in the literature hence other studies looking into using the coracoid process and the acromion as bony landmarks with a greater sample size of cadavers or studies using radiological investigations may be needed to increase the confidence in the findings on this project.

As stated previously, the coracoacromial plane demarcated the 2nd part of the axillary artery as a safe place to carry out cannulation. This finding, however, is not quite in line with other studies such as the study carried out by Dawson et al. [5], which offers an iteration of the way in which cannulation of the axillary artery should occur. Dawson et al. [5] state that the first part of the axillary artery is the ideal location for cannulation so that vascular compression against the second rib can occur and to avoid many of the branch vessels thereby reducing the risk of haematoma formation. This was also reasoned in the paper by Cheney and McCabe [12].

It is important to note that the coracoacromial plane can be affected by various aspects but most importantly patient arm position. Since the acromion and the coracoid are part of the scapula, their positions are affected by movements that involve the scapula such as arm adduction, abduction, shoulder elevation, and depression, just to name a few [13]. The study was carried out in a neutral cadaveric position with the arms of the cadavers adducted maximally and shoulders in a neutral position; however, this was not possible for all the cadavers since after the embalming procedure, the manipulation of the limbs into a neutral position was not always feasible, which may affect the trajectory of the coracoacromial plane.

The study also calculated the area of safety in regard to the cannulation procedure, which helped to give insight into the margin of error that can be allowed for using the techniques outlined in this study as well as inform clinical decisions regarding the preferred side of cannulation in the case of the area of safety being larger than on the left or vice versa. Due to this being a novel concept, there is no prior research within this realm. However, a big aspect of calculating area involved measuring the diameter of both the right and left axillary artery for which there is a wide array of literature available. Since the branching patterns of the arteries supplying the upper limb differ on the left and right sides of the body, it can be hypothesised that the diameter between the left and right axillary arteries may differ with the diameter being wider on the left side compared to the right. However, this study found that the mean diameter of the axillary artery on the left side for all the cadavers used in the study (n = 9) was 9.04 mm and on the right side (n = 8) it was 8.83 mm. Despite the right-sided diameter being slightly smaller than the left-sided diameter, there was not a statistically significant difference observed between the two sides as confirmed by carrying out the Student's t-test, which resulted in a p-value of 0.31, which is greater than 0.05, which indicates that there was no statistical significance between the left and the right side despite the branching patterns in place. This small difference may be related to the fact that cadaver 1 only had the left side dissected meaning that right-sided measurements were not performed resulting in an uneven sample size between the left and right. Bearing in mind the non-significant differences in diameters, a non-significant difference for the area of safety was also seen with the Student's t-test generating a p-value of 0.62, which is greater than 0.05, indicating no significant difference.

In the study carried out by Tayal et al. [14], the diameter of both the right and left axillary artery was measured via a CT angiography of the artery in which the mean results were calculated as being 6.52 mm in the left and 6.38 mm in the right, which are smaller than the results obtained from this study. There could be a number of reasons as to why this might be the case, for example, a larger sample size of 110 patient scans was included in the study, which may result in more reliable results. Furthermore, the use of CT scans could increase the accuracy and reliability of establishing measurements of the diameter as the chance of human error is reduced. Tayal et al. [14] also do not specify whether the diameter measured in their study was the internal luminal diameter (which is more likely to undergo age-related pathological changes) or the external diameter (involving the thickness of the vessel wall), which was the case in this project. Additionally, Tayal et al. [14] do not specify which part of the axillary artery was the diameter measured from as this may affect the diameter size.

A study by Schäfer et al. [15] investigated the safety and efficacy of percutaneous transaxillary access for heart valve replacement and found that the mean minimum axillary artery diameter in surgical patients was 6.85 mm ± 0.17. Whilst it is not clear what the term ‘minimum’ axillary diameter means, the article suggests that this was the intraluminal diameter measurement, which excluded the vessel wall and could suggest the difference between the results from this project and the study by Schäfer et al. [15]. Results from previous studies [15,14], however, are very similar, which could be the result of using radiological imaging to determine the diameter of the vessel, which lends itself more accurately compared to using callipers.

Arnett et al. [16] also looked at the measurements of the axillary artery diameter using a CT angiogram. The study concluded a mean measurement of the minimum luminal diameter of 6.0 ± 1.1 mm, which is fairly close to the other radiological studies mentioned above. However, since this was the luminal diameter, it was therefore smaller than the mean diameter obtained in this project. The wall of an artery is composed of three main layers: the inner intimal layer, the middle tunica layer, and the outer adventitia. Various studies have been carried out that have investigated the thickness of vessel walls. One such study carried out by Martire et al. [17] reports that the intima-media thickness (IMT) of the axillary artery is expected to be 1 mm. Anything above this indicates pathology. Adding this to the internal diameter measurements that have been obtained in the above studies brings the diameter closer to what was obtained in this project; however, it still goes without saying that radiological measurement of the diameter is far superior to manual measurements using callipers.

Limitations

The project had limitations that should be considered when thinking about drawing conclusions from the results. Firstly, due to the limited sample size of the study, adequate statistical power was not met. Carrying out statistical power calculations helps determine a minimum sample size that would be required to ensure that the study has enough power to detect differences between groups and therefore comment on statistical significance. The higher the sample size of a given study, the higher the statistical power will also be [18]. Having a high power increases the chances that the results will show differences between groups if there is one, i.e., between the left and right-sided measurements carried out in this project. Therefore, although there were no significant differences between any of the results obtained in this project, it may be due to the lack of a big enough sample size as the minimum sample size to detect a difference may have not been met.

Secondly, although a total of nine cadavers were used in this project, statistical analysis was only able to be carried out on cadavers 2-9 since cadaver 1 was only dissected unilaterally on the left side as it was only a cadaver used for teaching purposes. Therefore, there are more datasets for the left side compared to the right side in this project, which may skew data such as the mean for the left-sided measurements. What is more is that data on patient co-morbidity were not present and therefore were not accounted for during the analysis of the results.

Furthermore, this project may have benefitted from a second observer carrying out the methodology to calculate inter-observer differences. Since the end goal of this project is to identify bony landmarks that can be used to aid in safe cannulation of the axillary artery, it would have been beneficial to investigate the ability of another person to follow the outlined method using the bony landmarks suggested and see whether statistically similar results are obtained between two different researchers. Furthermore, intra-observer differences would have also been beneficial to see whether similar results are obtainable across different points of time, all of which would have helped to increase confidence in the reproducibility of the method.

Also, due to the embalmed nature of the cadavers, there is a limitation on how much the limbs and shoulders can be manipulated into different positions, i.e., shoulder position. This is something that can influence the results in this project since elevation of the shoulders can lead to overestimation of the distances measured and shoulder depression may lead to underestimates. A lack of range of motion is most notably seen with deceased donors that have been embalmed using formalin [19] and this was heightened since the cadavers used in this project were embalmed two years prior to the commencement of this project.

Due to the cadavers used in this project being used for teaching for 1st and 2nd-year medical students, the axillary region was superficially dissected by these students prior to starting the project. Due to the variability in the dissection of this region amongst students, there were often discrepancies in the integrity of anatomical structures present; for example, the brachial plexus and axillary sheath were damaged or overhandled, which could have led to some unreliable measurements in this region. An important consideration to make is the role of the axillary sheath. In a clinical setting, the axillary sheath would cause soft tissue structures like the brachial plexus and axillary artery to lie in even closer proximity to each other. Since the axillary sheath was dissected in this project, the anatomical structures would have been spaced out more hence affecting the external validity of the results obtained. Although care was taken to reposition the neurovascular contents as naturally as possible, encasement within the connective tissue sheath would increase the complexity of the procedure in living patients; therefore further studies to investigate this in greater depth are needed.

## Conclusions

Continuously innovating new techniques, approaches, and methods in medical care is imperative to help advance modern medicine. Endovascular procedures carried out via the femoral artery are one such example where modern medicine has evolved to make surgical procedures safer and more accessible to a wider array of patients. Extending this even further, the axillary artery has also been proposed as an alternative route for cannulation, particularly in those patients who may suffer from peripheral arterial disease or have hostile anatomy of the vasculature of the lower limb. The results from this project have highlighted the acromion and the coracoid process can be used in a novel way as two bony landmarks to define a trajectory that may aid in the safe cannulation of the axillary artery as this coracoacromial plane consistently traverses the axillary artery whereby the cords of the brachial plexus, the median nerve, and thoracoacromial trunk can be avoided. This therefore helps to tackle many of the complications that are found in literature regarding haematoma formation secondary to branch cannulation and brachial plexus injuries.

However, further research is needed in this field and a larger study using the coracoacromial plane made using the acromion and coracoid process should be carried out using radiological techniques on living patients. This would ensure that the anatomy of the axillary region would not have been displaced by dissection leading to a more accurate and reliable dataset. Overall, preliminary findings from this project look promising to support further research in this field.
